# Effects of narasin supplementation frequency on intake, ruminal fermentation parameters, and nutrient digestibility of *Bos indicus* Nellore steers fed with forage-based diets

**DOI:** 10.1093/tas/txab125

**Published:** 2021-07-21

**Authors:** Letícia Carolina Bortolanza Soares, Rodrigo S Marques, Alexandre Vaz Pires, Vinicius Alves Cruz, Arnaldo Cintra Limede, Kauê dos Santos Maia, Marcelo Baggio, José Paulo Roman Barroso, Janaina Sokolovski Biava, Evandro Maia Ferreira, Marcos Vinicius de Castro Ferraz Jr, Daniel Montanher Polizel

**Affiliations:** 1 Department of Nutrition and Animal Production, FMVZ, University of São Paulo, Pirassununga, SP 13635-000, Brazil; 2 Department of Animal Science, College of Agriculture “Luiz de Queiroz”, University of São Paulo, Piracicaba, SP 13418–900, Brazil; 3 Department of Animal and Range Sciences, Montana State University, Bozeman, MT 59717, USA; 4 Department of Animal Science, Federal University of Amazonas, Parintins, AM 69152–240, Brazil

**Keywords:** digestibility, feed additives, forage, narasin, ruminal parameters, supplementation frequency

## Abstract

The study aimed to evaluate if the frequency of narasin supplementation impacts dry matter intake, ruminal fermentation parameters, and apparent digestibility of nutrient in Nellore (*Bos indicus*) steers fed forage-based diets. A total of 32 rumen-cannulated Nellore steers (initial body weight [BW] = 317 ± 27 kg; age =18 ± 1 mo) were assigned to individual pens in a randomized complete block design according to their initial shrunk BW. Within block, steers were randomly assigned to 1 of 4 treatments: 1) forage-based diet without the addition of narasin (CON; *n* = 8), 2) CON diet plus 13 ppm of narasin every 24 h (N24; *n* = 8), 3) CON diet plus 26 ppm of narasin every 48 hours (N48; *n* = 8), or 4) CON diet plus 39 ppm of narasin every 72 hours (N72; *n* = 8). The experimental period lasted 30 d, with 18 d for diet adaptation and 12 d for sample collection. The experimental diets contained 95% of Tifton-85 (*Cynodon dactylon* spp.) haylage and 5% ground corn used as a delivery vehicle for narasin. Ruminal fluid was obtained from d 25 to 30 at 6 h after feeding to determine ruminal fermentation parameters. Narasin supplementation frequency did not affect (*P* ≥ 0.22) nutrient intake and total tract apparent digestibility. Steers fed N24 and N48 had reduced (*P* = 0.02) ruminal acetate concentration compared with CON and N72. Daily supply of narasin increased (*P* = 0.01) the molar proportion of propionate compared with CON and N72, and it did not differ between N24 vs. N48, N48 vs. N72, and N72 vs. CON. Also, N48 steers had greater (*P* = 0.01) rumen propionate concentration compared with CON. The N24 treatment decreased the Ac:Prop (*P* = 0.01) and AcBut:Prop (*P* = 0.02) ratio compared with CON and N72, while N48 had reduced (*P* = 0.01) Ac:Prop and AcBut:Prop ratio when compared with CON steers. Steers fed N24 and N48 had greater (*P* = 0.04) ruminal short-chain fatty acids compared with CON, but it did not differ (*P >* 0.11) between N24, N48, and N72. Supplementing narasin to steers fed forage-based diets decreased (*P* < 0.01) ruminal ammonia concentration compared with CON steers regardless of supplementation frequency, being the least result observed for N24 steers. Collectively, narasin supplementation frequency affected fermentation parameters without altering the nutrient intake and total tract apparent digestibility. Hence, decreasing frequency of narasin supplementation to Nellore steers fed a forage-based diet did not reduce the capacity to modulate rumen fermentation parameters.

## INTRODUCTION

Feed additives are an important aspect of dietary management to enhance feed efficiency and profitability in grazing beef cattle systems (Berchielli and Bertipaglia, 2010) by altering rumen environment and fermentation routes and increasing energy and nitrogen efficiency metabolism (Tedeschi et al., 2003). Ionophores are the most studied and used feed additives in cattle, mainly for optimizing rumen fermentation and reducing the rates of digestive disorders (Ladeira et al., 2014). Nevertheless, the proportion of studies evaluating the use of ionophores in ruminants fed high-forage diets is limited compared with studies evaluating high-concentrate diets (Goodrich et al., 1984; Tedeschi et al., 2003).

In grazing-based systems, supplementation strategies (mineral, protein, and energy) are often adopted to minimize the unbalanced nutrient composition of the forage to meet the animal nutrient requirement (McDowell and Arthington, 2005; NASEM, 2016). Low-consumption supplements are the most feasible and simple alternative in grazing systems and often serve as carriers for feed additives (McDowell, 1996; Bretschneider et al., 2008). Although ionophores have been shown to enhance productivity of beef cattle fed forage-based diets (Bretschneider et al., 2008), their use is limited, given reduced supplement intake, increased intake variability across animals and over time, and labor required for their utilization in grazing systems (Davenport et al., 1989; Bretschneider et al., 2008; Cappellozza et al., 2019). Furthermore, the infrequent supplement intake by grazing animals (Cappellozza et al., 2019) might impact the effects of a feed additive on rumen metabolism and growth performance (Bretschneider et al., 2008). Large meal size may also increase the probability of feed additive toxicity in grazing animals, if the bunk space is not appropriate to avoid overconsumption (Horn, 2006). Hence, it is crucial to establish if decreasing supplement frequency with feed additives would impact rumen metabolism in grazing beef cattle.

Narasin is an ionophore that improves performance of grazing beef cattle without affecting mineral (Silva et al., 2015) or protein supplement intake (Polizel et al., 2019). Daily supplementation of narasin to beef cattle fed forage-based diets increased concentration of ruminal propionate and total short-chain fatty acids (SCFA) and reduced acetate:propionate (Ac:Prop) ratio (Miszura et al., 2019; Polizel et al., 2020; Limede et al., 2021). Also, supplementing narasin daily into low-consumption mineral and protein supplements increased animal performance (Polizel et al., 2017, 2018, 2019), given these supplements are often consumed erratically by animals (Cappellozza et al., 2019), denoting a possible lasting effect of this molecule on ruminal metabolism. Accordingly, Pasqualino et al. (2020) reported an increased rumen propionate concentration even after 4 days of narasin withdrawal, suggesting a lasting residual effect of narasin on rumen fermentation routes. Based on this rationale, we hypothesized that infrequent narasin supplementation to beef cattle will not affect dry matter intake (DMI), nutrient digestibility, and ruminal fermentation parameters. Hence, our objective was to determine the effects of narasin supplementation frequency on DMI, ruminal fermentation parameters, and nutrient digestibility of *Bos indicus* Nellore steers fed a forage-based diet.

## MATERIALS AND METHODS

This experiment was conducted at the University of São Paulo, Piracicaba campus (USP/ESALQ; Piracicaba, SP, Brazil; 22°43′31″ S, 47°38′51″W, and 524 m elevation). Experimental procedures involving animals were reviewed and approved by the Ethics Committee on the Use of Animals at the same institution (University of São Paulo; protocol #5270260520).

### Animals, Experimental Design, and Diets

A total of 32 rumen-cannulated Nellore steers (initial body weight [BW] = 317 ± 27 kg; age = 18 ± 1 mo) were assigned to individual pens (2.5 × 4.5 m; concrete surface, with a feed bunk and waterer) in a randomized complete block design according to their initial shrunk BW. Within block (*n* = 8), steers were randomly assigned to 1 of 4 treatments: 1) forage-based diet without the addition of narasin (CON; *n* = 8), 2) CON diet plus 13 ppm of narasin (Zimprova; Elanco Animal Health, Sao Paulo, Brazil; N24; *n* = 8) every 24 h, 3) CON diet plus 26 ppm of narasin every 48 h (N48; *n* = 8), or 4) CON diet plus 39 ppm of narasin every 72 h (N72; *n* = 8). The administration rates of narasin used herein were according to the manufacturer’s recommendation. The experiment consisted of a 30 d period with 18 d for diet adaptation and 12 d for sample collection.

Throughout the experimental period (d 0 to 30), steers were offered 95% of Tifton-85 haylage (*Cynodon dactylon* spp.) and 5% of ground corn, which was used as a delivery vehicle for narasin treatments (N24, N48, and N72). Haylage was chopped daily with a vertical mixer (Mixer VM8B, DeLaval International AB, Rumba, Sweden). The concentrate was offered to each steer individually and daily before haylage feeding to avoid that the small amount of supplement would be mixed with hay and compromise the immediate intake of the mixture. On days when the N48 and N72 steers were not supplemented with narasin, the CON treatment concentrate (without feed additive) was offered to maintain similar forage:concentrate ratio between treatments. It is important to note that the treatments rate used herein aimed to provide 13 ppm of narasin daily. Treatment amounts were calculated based on the previous day individual total forage DMI. Steers were fed the concentrate once daily (0800 h) and followed by haylage (0830 h). Steers promptly consumed concentrate within 30 min after feeding. All steers had ad libitum access to haylage, fresh water, and mineral mix (offered in separately feed bunk from the haylage and concentrate) for the entire 30-d period. The mineral mix (Premiphós 80; Premix; Ribeirão Preto, SP, Brazil) used herein contained 150 g/kg Ca, 80 g/kg P, 12 g/kg S, 134 g/ kg Na, 4,500 mg/kg Zn 1,600 mg/kg, 1,400 mg/kg Mn, 800 mg/kg F, 210 mg/kg Co, 180 mg/kg I, and 27 mg/kg Se. The nutritional profile of the haylage and concentrate (ground corn) are described in Table 1.

**Table 1.** Nutritional profile of the Tifton-85 (*C. dactylon* spp.) haylage and ground corn^*a*^^,^^*b*^

Item	Haylage	Corn
Nutrient profile, dry matter basis		
Dry matter	47.5	88.0
Crude protein, %	20.8	9.10
Neutral detergent fiber, %	63.3	13.3
Acid detergent fiber, %	30.6	3.90
Hemicellulose, %	32.7	9.4
Ether extract, %	2.58	4.0
Ash, %	9.22	1.64
Total digestible nutrients^*c*^, %	55.1	88.6
Digestible energy, Mcal/kg	2.43	3.91
Metabolizable energy^*d*^, Mcal/kg	1.99	3.20
Net energy of maintence^*d*^, Mcal/kg	1.14	2.20
Net energy of gain^*d*^, Mcal/kg	0.59	1.52

^*a*^Based on nutritional profile of each ingredient, which were analyzed via wet chemistry procedures (AOAC, 1990).

^*b*^The experiment consisted of a 30 d period with 18 d for diet adaptation and 12 d for sample collection.

^*c*^Calculations were performed according to the equations proposed by Weiss et al. (1992).

^*d*^Calculated composition using tabular values from NASEM (2016).

### Sampling, Laboratory Analyses, and Measurements

Samples of haylage and concentrate were collected weekly, pooled across all weeks, and analyzed for nutrient profile (Table 1). From d 19 to 24, total fecal production was collected and quantified from the ground twice a day at 0800 h and 1800 h using an electronic scale (Marte AC-10K; Marte Cientifica, Sao Paulo, SP, Brazil), and a representative sample (approximately 10% of wet weight) of the daily production of each steer was collected and stored at −18°C on the same day of collection. Pens used to allocate the animals in this experiment were built with a 1% slope to separate and minimize urine contamination in the feces sample. Total tract apparent nutrient digestibility was calculated according to the formula: TTAD (%) = ([DMI × NCDM] − [FDM × NCFM] × 100)/(DMI × NCDM), where TTAD is the total tract apparent digestibility, DMI is the dry matter intake, NCDM is the nutrient content of the DMI (%), FDM is the fecal dry matter, and NCFM is the nutrient content of the fecal DM (%).

Samples of haylage, concentrate, orts, and feces were thawed, dried in a forced-air oven at 55°C for 96 h (AOAC, 1990; method #930.15), and ground through a 1-mm Wiley Mill screen (Marconi, Piracicaba, SP, Brazil). Dry matter content was determined by oven-drying the samples at 105°C for 24 h (AOAC, 1990); #934.01). Ash was determined by incinerating the samples in a muffle furnace at 550°C for 4 h (AOAC, 1990). Total nitrogen (N) concentration was determined using a LECO TruMac N Analyzer (Leco Corporation, St. Joseph, MI; AOAC, 1990; method #968.06). Crude protein (CP) was calculated by multiplying the total N content by 6.25. Neutral detergent fiber (NDF) was determined according to Van Soest et al. (1991), using an Ankom 2000 fiber analyzer (Ankon Tech. Corp., Macedon, NY). Acid detergent fiber (ADF) was determined according to Goering and Van Soest (1970). The NDF and ADF concentration were ash corrected, and sodium sulfite and heat-stable α-amylase were added in the NDF analysis. Hemicellulose was calculated based on the difference between the NDF and ADF values. The ether extract content was determined using an Ankom^XT15^ extractor (Ankon Tech. Corp.) according to AOAC (1990; method #920.29), using petroleum ether. Calculation of haylage and concentrate total digestible nutrients, net energy for maintenance (NE_m_), and gain (NE_g_) was performed according to Weiss et al. (1992) and the tabular values proposed by NASEM (2016).

Individual shrunk BW was collected on d 0 and 31 after 14 h of feed and water withdrawal to determine initial and final BW and to perform the randomization into blocks and treatments. Forage, concentrate, and total DMI were recorded daily from each pen by collecting and weighing non-consumed feed (forage only). Samples of the offered and non-consumed feed were collected daily from each pen and dried for 24 h at 105 ± 2°C in forced-air ovens for dry matter calculation.

From d 25 to 30 of the experimental period at 6 h after feeding, ruminal fluid samples were manually collected (approximately 100 mL/sample time) by squeezing the ruminal contents into four layers of cheesecloth, and the ruminal fluid pH was immediately determined (Digimed-M20; Digimed Instrumentação Analítica; São Paulo, SP, Brazil). Approximately 50 mL of the ruminal fluid were collected and stored at −18°C for subsequent analysis of rumen ammonia and molar proportions of individual SCFA (acetate, propionate, butyrate, isobutyrate, valerate, and isovalerate), as well as the acetate:propionate (Ac:Prop) and acetate butyrate:propionate (AcBut:Prop) ratios, and total SCFA. Frozen ruminal samples (1.6 mL) were prepared for analysis by thawing, centrifuging (15,000 × *g*) for 60 min at 4°C, and analyzed for SCFA and rumen N-NH_3_ concentration according to procedures described by Ferreira et al. (2016) and Broderick and Kang (1980), respectively.

### Statistical Analysis

The data were analyzed using the MIXED Procedure (SAS Inst., Inc., Cary, NC). For all the variables analyzed, animal was considered the experimental unit. The data were analyzed for normality of residues, homogeneity of variances, and removal of outliers. All data were analyzed using Kenward–Roger approximation to determine the denominator df for the test of fixed effects, with animal(treatment) as a random variable. Model statement for all analyses contained the effects of treatment, day, and treatment × day interactions, except for nutrient intake and digestibility variables that used the effects of treatment. In addition, block was used as an independent covariate for all analyses. The specified term for all repeated statements was day, with animal (treatment) as a subject. The covariance structure used was first-order autoregressive, which provided the smallest Akaike information criterion and hence the best fit for all variables analyzed. All results are reported as least square means and separated using PDIFF. Significance was set at *P* ≤ 0.05 and tendencies were determined if *P* > 0.05 and ≤ 0.10. Results are reported according to the main effects if no interactions were significant.

## RESULTS

Based on the manufacturer’s recommendation (13 ppm per day) and previous day total forage intake, narasin consumption during the experimental period were 13.7 ± 0.2, 13.4 ± 0.1, and 13.5 ± 0.1 mg/kg of DM per day for N24, N48, and N72, respectively. Decreasing frequency of narasin supplementation did not affect (*P* ≥ 0.51) DMI and specific nutrient intake (Table 2). No treatment effects were detected (*P* ≥ 0.22) for total apparent nutrient digestibility of steers fed forage-based diet.

**Table 2.** Intake and apparent total tract digestibility of nutrients of *B. indicus* Nellore steers receiving a forage-based diets supplemented or not (CON; *n =* 8) with narasin every 24 h (N24; *n =* 8), 48 h (N48; *n =* 8), or 72 h (N72; *n =* 8)

Item	Treatments^*a*^				SEM	*P*-value
	CON	N24	N48	N72		
Intake, kg/day						
Dry matter	4.79	4.54	4.91	4.86	0.20	0.51
Organic matter	4.34	4.10	4.44	4.39	0.18	0.51
Crude protein	0.96	0.90	0.98	0.97	0.04	0.53
Neutral detergent fiber	2.88	2.70	2.93	2.91	0.13	0.52
Acid detergent fiber	1.38	1.29	1.40	1.40	0.06	0.52
Hemicellulose	1.50	1.41	1.53	1.51	0.07	0.52
Digestibility, % (dry matter basis)^*b*^						
Dry matter	63.2	62.3	65.3	62.8	1.30	0.38
Organic matter	65.4	64.7	67.5	65.3	1.18	0.39
Crude protein	67.0	67.3	69.8	67.1	0.90	0.22
Neutral detergent fiber	67.1	65.0	67.7	67.1	1.62	0.51
Acid detergent fiber	66.1	63.8	66.6	66.2	1.89	0.51
Hemicellulose	68.1	66.1	68.7	67.9	1.42	0.54

^*a*^CON, forage-based diet without the addition of narasin; N24, CON diet plus 13 ppm of narasin (Zimprova; Elanco Animal Health, Sao Paulo, Brazil) every 24 h; N48, CON diet plus 26 ppm of narasin every 48 h; N72, CON diet plus 39 ppm of narasin every 72 h.

^*b*^From d 19 to 24, total fecal production was collected and quantified twice a day at 0800 h and 1800 h to determine total tract apparent nutrient digestibility analysis. Apparent digestibility was calculated according to the formula: TTAD (%) = ([DMI × NCDM] − [FDM × NCFM] × 100)/(DMI × NCDM), where TTAD, total tract apparent digestibility; DMI, dry matter intake; NCDM, nutrient content of the DMI (%); FDM, fecal dry matter; and NCFM, nutrient content of the fecal DM (%).

No treatment × day interaction was detected (*P* ≥ 0.11) for ruminal fermentation parameters (Table 3). A treatment effect was detected (*P* ≤ 0.04) for the molar proportion of acetate, propionate, and total SCFA as well as Ac:Prop and AcBut:Prop ratios in the ruminal fluid (Table 3). Steers fed N24 and N48 had reduced (*P* ≤ 0.05) molar proportion of acetate compared with CON and N72, whereas ruminal acetate did not differ (*P* > 0.86) between N24 vs. N48 and CON vs. N72. Conversely, the molar proportion of propionate was greater (*P* ≤ 0.04) in the ruminal fluid of steers fed N24 and N48 compared with CON and N72 steers, whereas ruminal propionate did not differ (*P* > 0.27) between N24 vs. N48, N48 vs. N72, and N72 vs. CON steers. Consequently, N24 and N48 steers had reduced (*P* ≤ 0.03) Ac:Prop and AcBut:Prop ratios compared with CON steers, whereas these ratios did not differ (*P* > 0.16) between N24 vs. N48, N48 vs. N72, and N72 vs. CON steers. No treatment effect was detected (*P* > 0.21) for molar proportion of isobutyrate, butyrate, isovalerate, and valerate (Table 3). Total SCFA did not differ (*P >* 0.11) between steers fed narasin regardless of frequency supplementation, whereas only steers fed N24 and N48 had greater (*P* < 0.01) molar proportion of total SCFA compared with CON steers (Table 3). A day effect was observed (*P* < 0.01) for all rumen variables herein analyzed (Table 3).

**Table 3.** Molar proportion of rumen SCFA, ammonia, and pH of *B. indicus* Nellore steers receiving a forage-based diets supplemented or not (CON; *n =* 8) with narasin every 24 h (N24; *n =* 8), 48 h (N48; *n =* 8), or 72 h (N72; *n =* 8)

Item	Treatments^*a*^				SEM^*b*^	*P-*value^*b*^		
	CON	N24	N48	N72		Treatment	Day	T × D
Short-chain fatty acids, mM/100mM^*c*^								
Acetate	74.27^a^	73.51^b^	73.38^b^	74.27^a^	0.30	0.01	<0.01	0.63
Propionate	14.44^c^	15.56^a^	15.20^ab^	14.80^bc^	0.24	<0.01	<0.01	0.36
Isobutyrate	1.28	1.22	1.20	1.27	0.04	0.19	<0.01	0.11
Butyrate	6.68	6.82	6.90	6.39	0.20	0.22	<0.01	0.79
Isovalerate	1.45	1.57	1.57	1.56	0.09	0.71	<0.01	0.65
Valerate	1.74	1.60	1.66	1.69	0.05	0.18	<0.01	0.95
Ac:Prop	5.16^a^	4.73^c^	4.85^bc^	5.05^ab^	0.10	<0.01	<0.01	0.37
AcBut:Prop^*d*^	5.63^a^	5.17^c^	5.31^bc^	5.48^ab^	0.10	0.01	<0.01	0.37
Total SCFA, mM	94.36^b^	104.47^a^	105.08^a^	97.13^ab^	3.02	0.04	<0.01	0.59
Rumen pH	6.79	6.70	6.68	6.79	0.05	0.17	<0.01	0.54
Ammonia, mg/dL	12.86^a^	9.33^c^	10.00^bc^	11.00^b^	0.76	<0.01	<0.01	0.23

^*a*^CON, forage-based diet without the addition of narasin; N24, CON diet plus 13 ppm of narasin (Zimprova; Elanco Animal Health, Sao Paulo, Brazil) every 24 h; N48, CON diet plus 26 ppm of narasin every 48 h; N72, CON diet plus 39 ppm of narasin every 72 h. Within rows, values with different superscripts differ (*P* ≤ 0.05). Ac:Prop, acetate:propionate ratio; AcBut:Prop, acetatebutirate:propionate ratio.

^*b*^*P* value for treatment, day and treatment × day interaction (T × D).

^*c*^From d 25 to 30 of the experimental period at 6 h after feeding, ruminal fluid samples were manually collected (approximately 100 mL/sample time) by squeezing the ruminal contents into four layers of cheesecloth, and ruminal fluid pH was immediately determined. Approximately 50 mL of the ruminal fluid were collected for analysis of rumen ammonia and molar proportions of individual SCFA according to procedures described by Ferreira et al. (2016) and Broderick and Kang (1980), respectively.

^*d*^Relationship between ketogenic and glucogenic volatile fatty acid in the rumen as reported by Polizel et al. (2020).

No treatment effect was detected (*P* = 0.17) for ruminal pH (Table 3). However, supplementing narasin to steers fed forage-based diets decreased (*P* < 0.01) ruminal ammonia concentration compared with CON steers regardless of supplementation frequency, being the least result observed for N24 steers (Table 3).

## DISCUSSION

Supplementation strategies (mineral, protein, and energy) in grazing cattle systems aim to minimize possible nutritional deficiencies of the forage to meet nutrient requirements of the animal (McDowell and Arthington, 2005; NASEM, 2016). Low-consumption supplements are the most feasible and uncomplicated alternative in grazing systems and often serve as carriers for feed additives (McDowell, 1996; Bretschneider et al., 2008). Nevertheless, supplementation programs considerably increase production costs in grazing cattle systems, including costs associated with supplement purchase and labor required for supplement feeding (Morais et al., 2014). One alternative to minimize labor and feeding costs in grazing systems is reducing the frequency of supplementation without affecting animal performance (De Paula et al., 2010; Moriel et al., 2016). Although feed additives have been used to enhance efficiency and growth of beef cattle (Tedeschi et al., 2003; Bretschneider et al., 2008; Duffield et al., 2012), their use in a low-consumption supplement offered infrequently is limited, given the potential reduction of supplement intake, intake variability across animals and over time, and labor required to apply this nutritional tool in grazing systems (Davenport et al., 1989; Bretschneider et al., 2008; Cappellozza et al., 2019). Also, decreasing supplementation frequency might affect meal size, increasing the probability of feed additive toxicity in grazing animals (Horn, 2006). Nevertheless, detailed studies investigating the frequency of feed additives supplementation on ruminal fermentation parameters are scarce and warranted investigation in grazing beef cattle.

Narasin is an antimicrobial ionophore that improves performance of grazing beef cattle without affecting mineral (Silva et al., 2015) or protein supplement intake (Polizel et al., 2019). Daily supplementation of narasin increased ruminal propionate and SCFA concentration and reduced Ac:Prop ratio in beef cattle fed forage-based diets (Miszura et al., 2019; Polizel et al., 2020; Limede et al., 2021). Additionally, daily supplementation of narasin into low-consumption mineral and protein supplements benefited performance in grazing cattle (Polizel et al., 2017, 2018, 2019), given these supplements are often consumed erratically by animals (Cappellozza et al., 2019), denoting a possible lasting effect of this molecule on ruminal metabolism. In fact, even after the removal of ionophores from the diet, there is still a residual effect on ruminal environment and fermentation parameters of steers consuming forage-based diets (Bell et al., 2017). These authors observed that the proportion of acetate remained lower and propionate remained greater up to 7 days after monensin withdrawal compared with non-supplemented steers (Bell et al., 2017). In the current study, decreasing frequency of narasin supplementation from daily to every 48 h did not affect propionate, acetate, and total SCFA as well as Ac:Prop and AcBut:Prop ratios, demonstrating a possible residual effect of this molecule in animals receiving forage-based diets. On the other hand, steers receiving narasin as infrequent as 72 h had intermediate values of these ruminal fermentation parameters and did not differ from the control group. Accordingly, our research group demonstrated a residual effect on ruminal environment when narasin was removed from the diet, resulting in a persisted greater proportion of propionate until day 4 after narasin withdrawal compared with unsupplemented animals (Pasqualino et al., 2020). These authors did not observe a residual effect on the proportion of acetate, whereas Ac:Prop ratio remained lower until day 3 after removing narasin from the diet (Pasqualino et al., 2020), corroborating with the results observed herein.

In grazing beef cattle, many factors affect the total concentration of SCFA in the ruminal fluid, including passage rate, water and saliva dilution, and production and absorption of acids (Leng and Brett, 1966). Recent data from our group also demonstrated that daily supplementation of narasin affects total ruminal SCFA concentration in steers fed forage-based diets (Polizel et al., 2020; Limede et al., 2021). In the present study, decreasing frequency of narasin supplementation from daily to every 48 h or 72 h did not affect the total concentration of SCFA, whereas the proportion of total SCFA was similar between steers receiving narasin infrequent as 72 h and unsupplemented steers. Moreover, a day effect was observed on all ruminal fermentation parameters, which might be attributed to the daily variation observed in quality, composition, and intake of the forage during the experiment period. Daily variations in forage quality and composition affect nutrient utilization and consequently ruminal fermentation parameters in animals fed forage-based diets (Hills et al., 2015; de Souza et al., 2017; Limed et al., 2021). Despite the differences in ruminal fermentation parameters observed herein, narasin supplementation could not affect the intake and apparent digestibility of nutrients regardless of frequency. Accordingly, Bell et al. (2017) reported no differences in nutrient digestibility of beef steers receiving a forage-based diet with or without monensin. Consistent with those findings, our research group also observed no differences in apparent digestibility of nutrients of *B. indicus* Nellore steers receiving forage-based diets with addition or not of narasin (Polizel et al., 2020; Limede et al., 2021). Hence, the present study demonstrates that decreasing the frequency of narasin supplementation from daily to every 48 h might be one alternative for beef cattle producers in defining supplementation strategies to optimize rumen fermentation parameters in grazing systems.

Forage-based diets typically have less concentration of rapidly fermentable carbohydrates, which stimulate the rumination process, increase the influx of saliva into the ruminal environment, and maintain ruminal pH close to optimal conditions (Galyean and Defoor, 2003). Corroborating with this statement, Osborne et al. (2004) proposed that for an ionophore to impact rumen pH, lactate concentration should exceed 5 mM, which was improbable in the present study. Research from our group and others also reported similar rumen pH of beef steers supplemented with narasin (Polizel et al., 2020; Limede et al., 2021) or monensin (Bell et al., 2017). Crossland et al. (2017) also reported similar rumen pH values of steers supplemented monensin in a forage-based diet. Hence, it is likely that ruminal pH values in the present study were kept in a range that would not impair rumen function (Yokoyama and Johnson, 1988), given that all animals consumed a forage-based diet and total tract digestibility was not affected by decreasing frequency of narasin supplementation.

The use of ionophores alleviates ruminal proteolysis, reduces ammonia synthesis, and increases the influx of protein into the small intestine (Goodrich et al., 1984; Rogers et al., 1997). In ruminants, rumen ammonia values below 5 mg/dL might limit microbial growth and ruminal fermentation parameters (Satter and Slyter, 1974; Slyter et al., 1979). In the present study, narasin supplementation reduced ruminal ammonia concentration by approximately 14%, 22%, and 27% when offered every 72 h, 48 h, or daily, respectively, compared with non-supplemented steers, suggesting that rumen function was not affected by the nutritional strategies adopted herein. Corroborating with our results, Pasqualino et al. (2020) reported a prolonged effect of narasin on the concentration of ammonia up to 3 d after the removal of the ionophore from diets. Hence, decreasing supplementation frequency of narasin up to every 72 h mitigated ruminal proteolysis and reduced ammonia synthesis in beef cattle offered forage-based diets.

Collectively, decreasing supplementation frequency of narasin from daily to every 48 h did not alter ruminal SCFA profile by impacting the molar concentration of acetate, propionate, butyrate, and total SCFA in steers fed forage-based diet. Additionally, supplementing narasin to beef steers fed a forage-based diet mitigated ruminal proteolysis and reduced ruminal ammonia concentration, regardless of supplementation frequency. However, narasin supplementation could not affect total nutrient intake and total tract apparent digestibility of nutrients, regardless of supplementation frequency adopted herein. Thus, the lasting effects of narasin might aid producers in defining supplementation strategies to obtain more economically efficient use of these molecules to optimize rumen fermentation characteristics and productivity in grazing beef cattle.

## References

[CIT0001] Association of Official Analytical Chemists (AOAC). 1990. Official methods of analysis, 12th ed. Washington (DC): AOAC.

[CIT0002] Bell, N. L., R. C. Anderson, T. R. Callaway, M. O. Franco, J. E. Sawyer, and T. A. Wickersham. 2017. Effect of monensin inclusion on intake, digestion, and ruminal fermentation parameters by *Bos taurus indicus* and *Bos taurus taurus* steers consuming bermudagrass hay. J. Anim. Sci. 95:2736–2746. doi:10.2527/jas2016.1011 28727060

[CIT0003] Berchielli, T. T., and L. M. A. Bertipaglia. 2010. Utilização de aditivos na produção de bovinos de corte. In: Bovinocultura de corte. v. 1. FEALQ, Piracicaba, SP. p. 295–323.

[CIT0004] Bretschneider, G., J. C. Elizalde, and F. A. Pérez. 2008. The effect of feeding antibiotic growth promoters on the performance of beef cattle consuming forage-based diets: a review. Livest. Sci. 114:135–149. doi:10.1016/j.livsci.2007.12.017

[CIT0005] Broderick, G. A., and J. H. Kang. 1980. Automated simultaneous determination of ammonia and total amino acids in ruminal fluid and in vitro media. J. Dairy Sci. 63:64–75. doi:10.3168/jds.S0022-0302(80)82888-8 7372898

[CIT0006] Cappellozza, B. I., P. V. F. Lasmar, F. T. Reis, L. Oliveira, F. Hoe, R. M. Boehler, J. Leibovich, R. Starkey, J. Simas, and R. F. Cooke. 2019. Effects of supplement type and narasin inclusion on supplement intake by *Bos indicus* beef bulls grazing a warm-season forage. Transl. Anim. Sci. 3:172–180. doi:10.1093/tas/txy113 PMC7200579 32704798

[CIT0007] Crossland, W. L., L. O. Tedeschi, T. R. Callaway, M. D. Miller, W. B. Smith, and M. Cravey. 2017. Effects of rotating antibiotic and ionophore feed additives on volatile fatty acid production, potential for methane production, and microbial populations of steers consuming a moderate-forage diet. J. Anim. Sci. 95:4554–4567. doi:10.2527/jas2017.1665 29108045

[CIT0008] Davenport, R. W., M. L. Galyean, M. E. Branine, and M. E. Hubbert. 1989. Effects of a monensin ruminal delivery device on daily gain, forage intake and ruminal fermentation of steers grazing irrigated winter wheat pasture. J. Anim. Sci. 67:2129–2139. doi:10.2527/jas1989.6782129x 2793626

[CIT0009] de Souza, J., F. Batistel, and F. A. P. Santos. 2017. Effect of sources of Ca salts of fatty acids on production, nutrient digestibility, energy balance, and carryover effects of early lactation grazing dairy cows. J. Dairy Sci. 100:1072–1085. doi:10.3168/jds.2016-11636 27939549

[CIT0010] De Paula, N. F., J. T. Zervoudakis, L. Da Silva Cabral, D. M. G. De Carvalho, L. K. Hatamoto-Zervoudakis, E. H. B. K. De Moraes, and A. A. De Oliveira. 2010. Supplementation frequency and proteins sources for growing of steers in pasture during the dry season: productive and economical performance. Rev Bras de Zootecn. 39:873–882. doi:10.1590/s1516-35982010000400024

[CIT0037] Duffield, T. F., J. K. Merrill, and R. N. Bagg. 2012. Meta-analysis of the effects of monensin in beef cattle on feed efficiency, body weight gain, and dry matter intake. J. Anim. Sci. 90:4583–4592. doi:10.2527/jas.2011-5018 22859759

[CIT0011] Ferreira, E. M., A. V. Pires, I. Susin, M. V. Biehl, R. S. Gentil, M. O. M. Parente, D. M. Polizel, C. V. D. M. Ribeiro, and E. Almeida. 2016. Nutrient digestibility and ruminal fatty acid metabolism in lambs supplemented with soybean oil partially replaced by fish oil blend. Anim. Feed Sci. Technol. 216:30–39. doi: 10.1016/j.anifeedsci.2015.09.007

[CIT0012] Galyean, M. L., and P. J. Defoor. 2003. Effects of roughage source and level on intake by feedlot cattle. J. Anim. Sci. 81:E8–E16. 10.2527/2002.8061395x 12078718

[CIT0038] Goering, H. K., and P. J. Van Soest. 1970. Forage fiber analyses (apparatus, reagents, procedures, and some applications), Agriculture Handbook No. 379ARS-USDA. Washington (DC): US Government Printing Office; p. 1–20.

[CIT0013] Goodrich, R. D., J. E. Garrett, D. R. Gast, M. A. Kirick, D. A. Larson, and J. C. Meiske. 1984. Influence of monensin on the performance of cattle. J. Anim. Sci. 58:1484–1498. doi:10.2527/jas1984.5861484x 6378865

[CIT0014] Hills, J. L., W. J. Wales, F. R. Dunshea, S. C. Garcia, and J. R. Roche. 2015. Invited review: an evaluation of the likely effects of individualized feeding of concentrate supplements to pasture-based dairy cows. J. Dairy Sci. 98:1363–1401. doi:10.3168/jds.2014-8475 25582585

[CIT0015] Horn, G. W. 2006. Growing cattle on winter wheat pasture: management and herd health considerations. Vet. Clin. North Am: Food Anim. Pract. 22:335–356. doi:10.1016/j.cvfa.2006.03.008 16814021

[CIT0016] Ladeira, M. M., O. R. M. Neto, L. de, C. Santarosa, M. L. Chizzotti, D. M. de Oliveira, J. R. R. de Carvalho, and M. C. L. Alves. 2014. Desempenho, características de carcaça e expressão de genes em tourinhos alimentados com lipídeos e monensina. Pesqui. Agropecu. Bras. 49:728–736. doi:10.1590/S0100-204X2014000900009

[CIT0036] Leng, R. A., and D. J. Brett. 1966. Simultaneous measurements of the rates of production of acetic, propionic and butyric acids in the rumen of sheep on different diets and the correlation between production rates and concentrations of these acids in the rumen. Br. J. Nutr. 20:541–552. doi:10.1079/BJN19660053 5923624

[CIT0017] Limede, A. C., R. S. Marques, D. M. Polizel, B. I. Cappellozza, A. A. Miszura, J. P. R. Barroso, A. S. Martins, L. A. Sardinha, M. Baggio, and A. V. Pires. 2021. Effects of supplementation with narasin, salinomycin, or flavomycin on performance and ruminal fermentation characteristics of *Bos indicus* Nellore cattle fed with forage-based diets. J. Anim. Sci. 99:1–11. doi:10.1093/jas/skab005 PMC8051843 33861855

[CIT0018] McDowell, L. R. 1996. Feeding minerals to cattle on pasture. Anim. Feed Sci. Technol. 60:247–271. doi: 10.1016/0377-8401(96)00983-2

[CIT0019] McDowell, L. R., and J. D. Arthington. 2005. Minerals for grazing ruminants in tropical regions. Mosaic. 4:86.

[CIT0021] Miszura, A. A., D. M. Polizel, J. P. R. Barroso, A. S. Martins, G. B. Oliveira, A. C. Limede, L. A. Sardinha, F. M. Neto, R. S. Marques, and A. V. Pires. 2019. Effects of feed additives on performance of yearling bulls fed high forage diet. J. Anim. Sci. 97:425–425. doi:10.1093/jas/skz258.843

[CIT0022] Morais, J. A. S., M. F. S. Queiroz, A. Keli, A. Vega, G. Fiorentini, R. C. Canesin, R. A. Reis, and T. T. Berchielli. 2014. Effect of supplementation frequency on intake, behavior and performance in beef steers grazing Marandu grass. Anim Feed Sci. Technol. 189:63–71. doi:10.1016/j.anifeedsci.2014.01.005

[CIT0023] Moriel, P., M. B. Piccolo, L. F. A. Artioli, M. H. Poore, R. S. Marques, and R. F. Cooke. 2016. Decreasing the frequency and rate of wet brewers grains supplementation did not impact growth but reduced humoral immune response of preconditioning beef heifers1. J. Anim. Sci. 94:3030–3041. doi:10.2527/jas.2015-0250 27482690

[CIT0024] NASEM (National Academies of Sciences, Engineering, and Medicine). 2016. Nutrient requirements of beef cattle. 8th Rev ed. Washington (DC): The National Academies Press.

[CIT0025] Osborne, J. K., T. Mutsvangwa, O. Alzahal, T. F. Duffield, R. Bagg, P. Dick, G. Vessie, and B. W. McBride. 2004. Effects of monensin on ruminal forage degradability and total tract diet digestibility in lactating dairy cows during grain-induced subacute ruminal acidosis. J. Dairy Sci. 87:1840–1847. doi:10.3168/jds.S0022-0302(04)73341-X 15453500

[CIT0026] Pasqualino, L. F., G. B. Oliveira, A. A. Miszura, J. P. R. Barroso, A. C. Limede, L. A. Sardinha, J. S. Biava, E. M. Ferreira, A. V. Pires, and D. M. Polizel. 2020. Residual effect of narasin on ruminal fermentation characteristics in lambs. Livest. Sci. 240:104141. doi:10.1016/j.livsci.2020.104141

[CIT0027] Polizel, D. M., M. J. P. T. Barbosa, B. I. Cappellozza, C. N. Lopes, M. V. C. F. Junior, L. G. M. Gobato, J. R. S. Gonçalves, and A. V. Pires. 2017. The addition of narasin into a mineral mixture improves performance of grazing Nellore steers. J. Anim. Sci. 95:267–267. doi:10.2527/asasann.2017.545

[CIT0028] Polizel, D. M., B. I. Cappellozza, F. Hoe, C. N. Lopes, J. P. Barroso, A. Miszura, G. B. Oliveira, L. Gobato, and A. V. Pires. 2020. Effects of narasin supplementation on dry matter intake and rumen fermentation characteristics of *Bos indicus* steers fed a high-forage diet. Transl. Anim. Sci. 4:118–128. doi:10.1093/tas/txz164 32704972 PMC7200564

[CIT0029] Polizel, D. M., M. V. C. Ferraz Jr., A. A. Miszura, J. P. R. Barroso, A. S. Martins, J. Goncalves, A. C. Limede, B. I. Cappellozza, E. M. Ferreira, A. V. Pires. 2018. Narasin improves performance of grazing Nellore yearling bulls. J. Anim. Sci. 96, Suppl 3, 447. doi: 10.1093/jas/sky404.976

[CIT0030] Polizel, D. M., A. C. Limede, A. A. Miszura, A. S. Martins, G. B. Oliveira, L. A. Sardinha, M. Baggio, A. V. Pires. 2019. Narasin improves performance of grazing Nellore yearlink bulls receiving protein supplement. J. Anim. Sci. 97, suppl 3, 421–422. doi: 10.1093/jas/skz258.835

[CIT0031] Rogers, M., J. P. Jouany, P. Thivend, and J. P. Fontenot. 1997. The effects of short-term and long-term monensin supplementation, and its subsequent withdrawal on digestion in sheep. Anim. Feed Sci. Technol. 65:113–127. doi:10.1016/S0377-8401(96)01089-9

[CIT0032] Satter, L. D., and L. L. Slyter. 1974. Effect of ammonia concentration on rumen microbial protein production in vitro. Br. J. Nutr. 32:199–208. doi:10.1079/bjn19740073 4472574

[CIT0039] Silva, R. G., M. V. Ferraz Junior, V. N. Gouveia, D. M. Polizel, M. H. Santos, A. A. Miszura, T. S. Andrade, M. F. Westphalen, M. V. Biehl, and A. V. Pires. 2015. Effects of narasin in mineral mix to Nellore heifers fed with high forage. J. Anim. Sci. 93(suppl S3):118.

[CIT0033] Slyter, L. L., L. D. Satter, and D. A. Dinius. 1979. Effect of ruminal ammonia concentration on nitrogen utilization by steers. J. Anim. Sci. 48:906–912. doi:10.2527/jas1979.484906x

[CIT0034] Tedeschi, L. O., D. G. Fox, and T. P. Tylutki. 2003. Potential environmental benefits of ionophores in ruminant diets. J. Environ. Qual. 32:1591–1602. doi:10.2134/jeq2003.1591 14535299

[CIT0040] Van Soest, P. J., J. B. Robertson, and B. A. Lewis. 1991. Methods for dietary fiber, neutral detergent fiber, and nonstarch polysaccharides in relation to animal nutrition. J. Dairy Sci. 74:3583–3597. doi:10.3168/jds.S0022-0302(91)78551-2 1660498

[CIT0035] Weiss, W. P., H. R. Conrad, and N. R. Pierre. St. 1992. A theoretically-based model for predicting total digestible nutrient values of forages and concentrates. Anim. Feed Sci. Technol. 39:95–110. doi:10.1016/0377-8401(92)90034-4

[CIT0041] Yokoyama, M. T., and K. A. Johnson. 1988. Microbiology of the rumen and intestine. In: D. C. Church, editor. The ruminant animal. New York (NY): Simon and Shuster; p. 125–144.

